# Supplementary material for: Comprehensive Analysis of Phenolic Constituents, Biological Activities, and Derived Aroma Differences of Penthorum chinense Pursh Leaves after Processing into Green and Black Tea
Source: Foods. 2024 Jan 26;13(3):399. doi: 10.3390/foods13030399 (PMC10855198; doi:10.3390/foods13030399)
Supplement: Supplementary file 1 [file foods-13-00399-s001.zip › foods-2798953-supplementary.pdf]

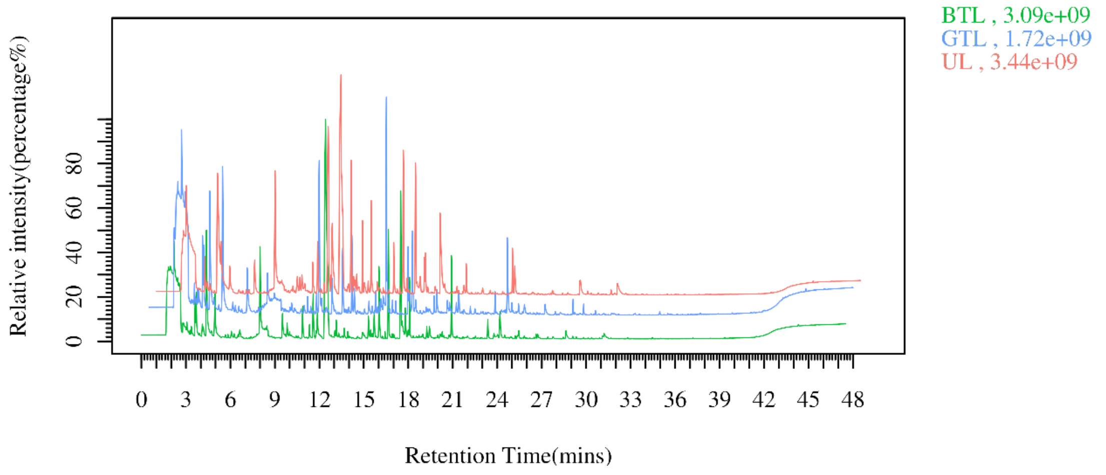

Figure S1. Typical total ion chromatograph (TICs) of *P. chinense* leaves (UL), *P. chinense* leaves green tea (GTL) and *P. chinense* leaves black tea (BTL).

Table S1. Volatile flavour compounds of *P. chinense* raw leaves (UL), green (GTL) and black tea (BTL)

Volatile flavour compound	Retention time (min)	CAS Number	Peak area (*10^7)		
			BTL	GTL	UL
Alcohols					
Ethanol	4.14	64-17-5	-	-	17315.93±707.11
1-Butanol	10.42	71-36-3	94.34±9.60	-	-
4-Heptenal, (Z)-	13.05	6728-31-0	270.98±8.07	-	-
1-Pentanol	13.67	71-41-0	408.90±46.75	1751.95±45.97	382.99±1.89
1-Hexanol	16.66	111-27-3	5100.74±231.72	240.00±18.78	9047.90±245.80
3-Hexen-1-ol	16.92	544-12-7	221.70±19.10	32.22±5.36	635.93±38.49
2-Hexen-1-ol, (E)-	18.08	928-95-0	2119.42±215.22	36.10±5.85	1645.51±284.13
1-Octen-3-ol	19.25	3391-86-4	408.06±27.63	411.96±22.60	1740.28±64.53
1-Hexanol, 2-ethyl-	20.28	104-76-7	240.97±27.04	224.93±12.65	262.53±35.22
Linalool	21.69	78-70-6	54.32±4.55	176.78±3.67	67.72±1.39

Terpinen-4-ol	23.01	562-74-3	-	-	21.10±0.07
2-Furanmethanol	24.36	98-00-0	-	9.34±1.74	-
2,6-Octadien-1-ol, 3,7-dimethyl-, (Z)-	28.53	106-25-2	-	16.57±1.67	-
1,6,10-Dodecatrien-3-ol, 3,7,11-trimethyl-	32.38	7212-44-4	-	5.62±0.46	-
3-Phenylpropanol	32.50	122-97-4	3.60±0.55	-	5.20±0.15
1-Tetradecanol	34.92	112-72-1	3.39±0.46	2.43±0.16	5.99±0.63
1-Hexadecanol	38.5317	36653-82-4	2.81±0.41	3.88±0.20	4.21±0.02
Aldehydes					
2-methyl-Propanal,	2.55	78-84-2	3583.24±97.36	3849.98±298.87	2747.22±147.10
Butanal	3.13	123-72-8	160.96±20.44	-	45.99±0.85
(E)-2-Butenal,	3.19	123-73-9	-	196.24±17.90	3.30±0.40
Pent-2-ynal	3.37	55136-52-2	339.69±1.11	413.07±24.64	369.72±14.54
2-methyl-Butanal,	3.63	96-17-3	1189.60±65.03	980.11±35.80	-

3-methyl-Butanal,	3.70	590-86-3	2264.60.±113.53	1524.51±179.20	181.97±4.51
2-methyl-Pentanal,	5.66	123-15-9	163.66±16.68	254.66±16.68	-
Hexanal	8.01	66-25-1	6249.84±728.12	1215.51±53.87	10085.67±303.61
2-Butenal, 2-methyl-	8.33	1115-11-3	706.15±29.39	272.85±44.15	-
3-Hexenal, (Z)-	9.99	6789-80-6	303.19±39.04	-	-
Heptanal	11.32	111-71-7	713.28±117.54	-	-
Octanal	14.57	124-13-0	131.71±16.45	161.33±12.08	-
2-Octenal, (E)-	18.51	2548-87-0	-	108.00±17.22	-
Decanal	20.37	112-31-2	128.13±10.75	97.70±1.83	-
Benzaldehyde	20.91	100-52-7	-	624.23±11.37	1740.89±240.84
(E,Z)-2,6-Nonadienal,	22.52	557-48-2	128.81±7.05	116.95±16.91	207.05±14.22
Benzeneacetaldehyde	23.80	122-78-1	406.60±27.08	170.87±3.89	90.04±2.96
1,3-Cyclohexadiene-1-carboxaldehyde, 2,6,6-trimethyl-	23.94	116-26-7	146.29±19.59	118.53±7.00	-

Cinnamaldehyde, (E)-	32.29	14371-10-9	-	1.30±0.12	-
Vanillin	41.47	121-33-5	2.42±0.38	-	9.21±1.25
Benzaldehyde, 4-hydroxy-	45.90	123-08-0	-	-	5.92±0.41
Ketones					
2-Pentanone	4.94	107-87-9	2614.26±258.18	-	-
Methyl Isobutyl Ketone	5.73	108-10-1	-	-	114.08±5.05
1-Penten-3-one	6.12	1629-58-9	107.55±11.00	-	-
3-Hexanone	7.09	589-38-8	-	51.97±9.28	-
2-Octanone	14.46	111-13-7	-	8.93±0.41	-
5-Hepten-2-one, 6-methyl-	16.04	110-93-0	3213.89±298.72	-	2368.80±282.71
2-Cyclohexen-1-one	18.64	930-68-7	31.00±5.43	-	27.35±4.06
Thujone	18.80	546-80-5	-	-	562.71±88.06
Bicyclo[2.2.1]heptan-2-one, 1,7,7-tri methyl-, (1S)-	20.75	464-48-2	-	-	628.48±6.52
Butyrolactone	23.46	96-48-0	-	-	43.56±4.20
Acetophenone	24.02	98-86-2	259.89±35.02	106.32±7.23	2015.15±186.42

2,6,6-Trimethyl-2-cyclohexene-1,4-dione	25.02	1125-21-9	10.07±0.88	9.58±1.43	-
3-Buten-2-one, 4-(2,6,6-trimethyl-1-cyclohexen-1-yl)-	30.37	14901-07-6	72.85±1.52	41.82±3.17	8.30±0.30
Esters					
Ethyl Acetate	3.28	141-78-6	146.20±13.86	344.41±37.91	1563.36±26.14
Formic acid, butyl ester	10.39	592-84-7	83.28±9.60	-	-
Hexanoic acid, ethyl ester	12.95	123-66-0	-	7.30±0.43	344.74±28.56
Acetic acid, hexyl ester	14.14	142-92-7	13.94±2.64	-	38.07±1.44
Cyclohexene, 3-methyl-6-(1-methylethylidene)-	14.28	586-63-0	22.19±2.17	82.77±15.92	605.65±14.45
cis-3-Hexenyl-**-methylbutyrate	19.74	53398-85-9	20.97±1.07	36.53±2.44	-
Formic acid, octyl ester	21.99	112-32-3	208.85±29.21	262.26±52.23	-
Benzoic acid, methyl ester	23.38	93-58-3	-	-	138.02±4.99
2(3H)-Furanone, 5-ethyldihydro-	25.21	695-06-7	125.31±16.74	64.97±11.40	47.33±0.62
cis-3-Hexenyl cis-3-hexenoate	25.73	61444-38-0	28.83±3.68	-	-
Methyl salicylate	26.86	119-36-8	-	-	27.93±1.16
Benzeneacetic acid, ethyl ester	27.13	101-97-3	-	-	5.77±0.17
Diisopropyl adipate	37.10	6938-94-9	-	13.27±1.58	-
Tetradecanoic acid, ethyl ester	32.54	124-06-1	-	-	0.94±0.06

Hexadecanoic acid, ethyl ester	36.34	628-97-7	-	-	11.05±0.59
Dimethyl phthalate	37.10	131-11-3	16.73±0.59	13.10±0.61	19.47±1.22
2(4H)-Benzofuranone, 5,6,7,7a-tetra hydro-4,4,7a-trimethyl-	37.88	15356-74-8	8.17±0.92	7.27±0.45	10.02±0.14
1,2-Benzenedicarboxylic acid, bis(2-methylpropyl) ester	41.17	84-69-5	-	-	2.83±0.40
Hydrocarbons					
Pentane	1.75	109-66-0	2436.85±6.90	-	2008.31±20.60
(1S)-2,6,6-Trimethylbicyclo[3.1.1]hept-2-ene	6.07	7785-26-4	464.59±12.48	194.32±5.18	270.40±23.23
Bicyclo[3.1.1]heptane, 6,6-dimethyl-2-methylene-, (1S)-	8.46	18172-67-3	-	-	519.53±38.82
Cyclohexene, 1-methyl-4-(1-methylethenyl)-, (S)-	11.60	5989-54-8	2275.06±96.48		16233.33±550.76
Styrene	13.51	100-42-5	-	171.99±7.14	1036.42±46.07
Ethane, 1,1,2-trichloro-	13.77	79-00-5	-	30.85±3.57	-
Tridecane, 3-methyl-	16.83	6418-41-3	-	-	46.44±9.63
Phenol, 2-methyl-5-(1-methylethyl)-	35.61	499-75-2	-	-	10.26±1.12

Phenol, 4-propyl-	44.20	645-56-7	-	-	17.87±1.10
Acids					
Butanoic acid, 3-methyl-	24.83	503-74-2	-	-	44.88±4.92
Butanoic acid	26.40	107-92-6	-	39.39±5.96	-
Heptanoic acid	28.70	111-14-8	-	102.91±11.98	-
Octanoic acid	33.07	124-07-2	24.95±4.11	29.33±3.15	43.66±4.97
Nonanoic acid	35.09	112-05-0	14.98±1.88	16.85±0.07	23.98±1.52
n-Decanoic acid	37.01	334-48-5	-	4.14±0.79	6.10±0.64
Dodecanoic acid	40.60	143-07-7	19.50±3.20	20.40±2.60	12.59±1.09
Oxacycles					
Furan, 2-ethyl-	4.39	3208-16-0	7805.76±511.80	687.75±9.59	6066.91±138.33
Furan, 2-pentyl-	12.80	3777-69-3	64.01±3.32	-	55.15±2.38
Ethanone, 1-(2-furanyl)-	20.50	1192-62-7	11.24±1.01	25.50±0.42	-

Benzothiazole	30.68	95-16-9	23.87±2.16	56.63±0.45	226.12±15.53
Other substances					
Ethanolamine	2.56	141-43-5	-	-	248.14±28.69
Furan, 3-methyl-	3.07	930-27-8	441.91±28.20	491.81±6.26	8.13±0.46
Dimethyl trisulfide	17.07	3658-80-8	-	47.59±4.00	-
Ammonium acetate	19.16	631-61-8	-	-	6513.33±635.33

- indicates that the compound is undetected or has a low match score.
